# In-House Validation of Four Duplex Droplet Digital PCR Assays to Quantify GM Soybean Events

**DOI:** 10.3390/foods13244011

**Published:** 2024-12-11

**Authors:** Daniela Verginelli, Sara Ciuffa, Katia Spinella, Davide La Rocca, Marisa Misto, Cinzia Quarchioni, Pamela Bonini, Cristiana Fusco, Lorella Peroni, Stefania Peddis, Ugo Marchesi

**Affiliations:** National Reference Laboratory for GM Food and Feed, GMO Unit, Istituto Zooprofilattico Sperimentale del Lazio e della Toscana “Mariano Aleandri”, 00178 Rome, Italy; daniela.verginelli@izslt.it (D.V.); sara.ciuffa@izslt.it (S.C.); davide.larocca@izslt.it (D.L.R.); marisa.misto@izslt.it (M.M.); cinzia.quarchioni@izslt.it (C.Q.); pamela.bonini@izslt.it (P.B.); cristiana.fusco@izslt.it (C.F.); lorella.peroni@izslt.it (L.P.); stefania.peddis@izslt.it (S.P.); ugo.marchesi@izslt.it (U.M.)

**Keywords:** GMO, validation, ddPCR, soybean, measurement uncertainty

## Abstract

Due to the increasing number of authorized events in the European Union, it is crucial for the official laboratories to enforce market control to detect and quantify genetically modified organisms. In this study, an in-house validation of quantitative duplex ddPCR methods was performed involving MON87701, MON87769, MON89788 and CV-127-9 assays with respect to the lectin reference gene. Since the ddPCR methods provide accurate quantification, show less sensitivity to PCR inhibitors and are more suitable for multiplexing compared to the real-time PCR, the optimization of the existing assays was performed with the exception of MON87701, according to the JRC Guidance documents and technical reports. However, some concerns related to practical settings for the quantitative multiplex of ddPCR methods and their validation were encountered; therefore, a general workflow to develop and validate a ddPCR-based method is shown. The obtained data and the validation performance parameters such as specificity, cross-talk, robustness, dynamic range, linearity, the limit of quantification, trueness and precision comply with international recommendations for GMO quantification methods. The duplex ddPCR methods here investigated are equivalent in terms of performance compared to the singleplex real-time PCR methods, showing higher flexibility and cost effectiveness.

## 1. Introduction

The biosafety aspects, regulations, and labelling for a genetically modified organism (GMO) and the derived food and feed products are rigorously regulated in most countries around the world [1,2,3]. The framework of the European Union demands event-specific methods for the detection, identification and quantification of GMO before being authorized and placed in the market [1,2]. Currently, several interesting approaches for genetically modified organism (GMO) detection and quantification have been implemented. The expansion and progress in the field of molecular diagnostic have granted appropriate calibrators as an anchor point for the measurement value and measurement units.

Most accepted methods for the detection and quantification of GMOs are DNA-based; among these the real-time polymerase chain reaction (qPCR) technology is widely used due to its higher sensitivity and robustness [4,5]. Despite qPCR efficiency, the quantification of the target and the reference gene can be affected by many factors, including inhibitors, and it is reliant upon standard curves for quantification [6,7,8,9].

To overcome such limits, in the last decade, the digital polymerase chain reaction (dPCR) has become an alternative to qPCR; indeed, different dPCR devices have been created, and a growing number of laboratories have transferred their validated qPCR testings into a dPCR format [10]. The ddPCR is more tolerable to the PCR inhibitors, as reported by Iwobi et al. [10], whose data validates the assumption that partitioning DNA into microreactions may lessen the impact of inhibitors on PCR amplification.

In the dPCR, the PCR mix containing the DNA target is partitioned in small compartments on a chip (chamber-based digital PCR or cdPCR), or the compartments correspond to water-in-oil droplets (droplet digital or ddPCR) [6]. In droplet digital PCR (ddPCR), the sample is partitioned into several thousands or millions of individual droplets in a water–oil emulsion, and subsequently, flow cytometry is used to count positive PCR tests [7]. In the ddPCR, unlike the qPCR, absolute single molecule detection allows GM event quantification without a standard curve, avoiding the amplification efficiency drawbacks observed with qPCR [8,9].

The detection of GMO plants is mainly based on PCR methods validated and published by the European Reference Laboratory for GM Food & Feed (EURL-GMFF) (European Reference Laboratory for GM Food & Feed https://gmo-crl.jrc.ec.europa.eu/method-validations, accessed on 25 January 2024). For routine applications in the scope of ISO 17025 [11], a full validation should be conducted to demonstrate the suitability of such methods. Several guidelines have already been released for the validation of PCR systems [6,12,13,14]. DdPCR protocols for the specific quantification of GM events, including several GM maize and soybean events and the respective reference genes, have been successfully established [15,16,17,18]. Nevertheless, the validation of ddPCR methods for the quantification of specific GM events has been occasionally reported [18,19,20]. Recently, Gatto et al. [19] reported a collaborative study to validate singleplex and duplex ddPCR assays to detect MON810 maize.

The objective of this work was to carry out an *in-house* validation to test the performance of the quantitative ddPCR duplex assay with respect to the lectin reference gene to detect MON87701, MON87769, MON89788 and CV-127-9 soybean. The measurement uncertainty (MU) determination, where the certified reference material was not available in the different GM levels, was another aspect investigated. In this study, the existing simplex qPCR EURL-GMFF validated methods [21,22,23,24] were transferred to the ddPCR platform, optimizing, where necessary, primer and/or probe concentrations to display clear discrimination between positive and negative signals according to the recommendations outlined in Pecoraro et al. 2019 [6]. DdPCR performance was assessed with regards specificity, cross-talk, robustness, dynamic range, linearity, the asymmetric limit of quantification (LOQ_asym_), accuracy (trueness and precision) and measurement uncertainty as reported in ENGL’s document [6,12,13,14,25].

## 2. Materials and Methods

### 2.1. Reference Material

The methods’ validation was performed on certified reference materials (CRMs) purchased from the American Oil Chemists’ Society (AOCS, Urbana, IL, USA). In particular, the following CRMs were used: MON87701 soybean (AOCS 0809-A2 ≥ 984 g/kg), MON87769 soybean (AOCS 0809-B2 ≥ 996 g/kg), MON89788 soybean (AOCS 0906-B2 ≥ 996 g/kg), CV-127-9 soybean (AOCS 0911-C2 ≥ 963 g/kg) and non-modified soybean (AOCS 0906-A < 0.8 g/kg and AOCS 0911-A ≤ 1.0 g/kg).

### 2.2. DNA Extraction and Assessment of DNA Purity

DNA was extracted from 150 mg of CRM powder by using the RSC PureFood GMO kit and the extractor Maxwell^®^RSC Instrument (Promega Madison, WI, USA), according to the manufacturer’s instructions. The concentration and the quality of the extracted DNA was directly assessed by ddPCR, evaluating the copy number of the lectin reference gene performing an inhibition test. The inhibition test was carried out in three serial dilution levels, with each level measured in duplicate [26]. The average of the absolute copies per reaction measured in the diluted samples multiplied by the dilution factor did not differ more than 25% from the average of the absolute copies per reaction measured on the highest concentration (lowest dilution) [14]. Dilutions of the extracted stock DNA solutions were made in nuclease-free water (Sigma-Aldrich Chemie GmbH, Munich, Germany) [27]. All of the DNA extracts were stored at +4 °C until further use.

### 2.3. Sample Preparation

Since the GM events considered in this work are available only at the 100% level, different GM levels of each event are produced mixing positive material (100% GM) with non-GM material. Starting from CRMs with 100% GM of MON87701, MON87769, MON89788, and CV-127-9, samples containing 10, 2, 1, 0.5, and 0.1 levels of GM soybean are produced as described in Hougs et al. (2017) (annex 3) [26]. Mixtures were prepared considering the concentration in the absolute copy number of the reference gene lectin (lec) measured by the ddPCR.

### 2.4. Specificity

The specificity of the duplex assay was in silico and experimentally assessed. The software Primer-dimer version 2018 http://www.primer-dimer.com/, accessed on 10 November 2024) was used for the prediction of oligonucleotide hybridization events including the resulting products and probes and self-dimers, while the Oligo-evaluator (http://www.oligoevaluator.com/LoginServlet, accessed on 1 February 2024) was used for cross-dimers and hairpin formation. In addition, the melting temperature (Tm) was analysed to verify the compatibility of the primers and probes of each duplex system. DNA melt curve analysis was carried out to verify the presence of an expected pure and single amplicon [28]. The PCR experiment was performed using a Rotor-Gene Q 5plex Platform (Qiagen, GmbH- Hilden, Germany) instrument in a final reaction volume of 20 μL, including the 1X FAST Eva Green qPCR Master Mix (Fisher Molecular Biology, Rome, Italy), 1 ng of genomic DNA, and 300 nM each of reverse and forward primers. Two technical replicates were performed for each sample. qPCR amplification was performed with the following programme: an initial denaturation of 2 min at 95 °C, followed by 40 cycles of 95 °C for 5 s, 60 °C for 30 s, and 72 °C for 20 s, with one fluorescence reading per annealing step. A post-PCR DNA melt curve analysis was carried out using a temperature ramping rate of 0.1 °C per step with a 30 s rest at each step.

### 2.5. Droplet Digital PCR and Analysis

The QX200 Droplet Digital PCR System (BioRad, Pleasanton, CA, USA) was used for the ddPCR method. The reaction volume was 20 μL containing 2X ddPCR Supermix for the probes (BioRad, Pleasanton, CA, USA) and 4 μL of template DNA. The ddPCR methods used in this work to detect the endogenous gene and GM events consist of an optimized version of previous methods in a real-time PCR published by EURL-GMFF [21,22,23,24]. Indeed, to increase the multiplexing capabilities of the method and achieve a clear cluster separation between positive signals and background noise, the primer and/or probe concentrations needed to be optimized. We select the optimal final concentrations of primers and probes as reported in Table S1.

The method to detect MON87701 represents an exception; indeed, the assay was the same as the qPCR without optimization. The PCR was carried out on a GeneAmp^TM^ PCR System 9700 thermocycler (Thermo Fisher Scientific, Waltham, MA, USA) under the following conditions: 10 min DNA polymerase activation at 95 °C, 45 cycles of a two-step thermal profile of 30 s at 94 °C for denaturation, 60 s at 60 °C for annealing and extension, droplet stabilization at 98 °C for 10 min, followed by an infinite hold at 4 °C. After thermal cycling, the 96-well plates were transferred to a QX200 droplet reader (Bio-Rad Laboratories, Inc.), and data were analysed with QX Manager 1.2 Standard Edition (Bio-Rad Laboratories, Inc.). According to the manufacturer’s instructions [29] and JRC’s documents [7], the data were accepted for the subsequent analyses if the number of droplets was more than 10,000 per 20 μL reaction with clear discrimination between positive and negative signals, i.e., if the negative controls (NTCs) were ≤2 fluorescent droplets and the positive control and sample were >2 fluorescent droplets.

### 2.6. Calculation of GM Content

After the ddPCRs were completed, the copy numbers for both event-specific and endogenous reference gene amplifications in each sample were calculated using the following equation:

(1) $$\mathrm{Concentration}=-\ln\;(\frac{Nneg}{N})/V_{\mathrm{droplet}}$$

where *Nneg* is the number of negative droplets; *N* is the total number of droplets; and V_droplet_ is the volume of droplets [30]. Finally, the GMO content in the mass fraction was calculated using the following equation reported in the application note of the European Union Reference Laboratory (EURL-GMFF) [30]:

(2) $$\mathrm{GM}\;\mathrm{content}\;(\%\frac{m}{m})=\frac{\left(cp\;GM\right)}{\left(cp\;taxon\;specific\;sequence\right)}\;\times \;\frac{1}{\left(CF\;GM\right)}\;\times \;100$$

where *cp GM* is the number of copies of the target per reaction, *cp* taxon-specific sequence is the number of copies of the lectin target per reaction, and *CF GM* is the conversion factor for the certified value of CRMs used as determined by the EURL GMFF (conversion factors (CFs)) for CRMs [30].

### 2.7. In-House Validation

The *in-house* validation study of GM soybean was assessed to evaluate specificity, cross-talk, robustness, dynamic range, linearity, the asymmetric limit of quantification (LOQ_asym_), accuracy (trueness and precision), and measurement uncertainty (MU). The specificity of the duplex assay was assessed as described previously. Precision was determined in terms of relative repeatability standard deviation (RSDr) obtained under repeatability conditions, while trueness was assessed in terms of relative bias. For the assessment of the dynamic range, linearity, trueness, precision and uncertainty measurements were analysed in six sample replicates in five runs of each event, resulting in 30 data points per each of the 6 GM levels. The detailed experimental design and technical approach of each parameter are described in Table 1.

### 2.8. Measurement Uncertainty

The MU was calculated according to the guidance for measurement uncertainty for GMO analysis [25] and the European technical guidance document for the flexible scope accreditation of laboratories quantifying GMOs [32].

MU is a single parameter that describes the quality of a measurement and the accuracy, taking into account all possible variabilities. Samples with GM levels 10%, 2%, 1%, 0.5% and 0.1% of MON87701, MON87769, MON89788 and CV-127-9 were prepared by mixing DNA extracted from 100% CRMs, with DNA extracted from non-GM soybean (NGM). The concentrations in terms of copies/µL were determined by ddPCR using the method for the taxon-specific lec target. The uncertainty was determined, firstly by calculating the GM mass fraction (wRM) of the reference material prepared in the laboratory following the formula described in Gatto et al. (2022) [19]:

(3) $$W_{RM}=\frac{C_{CRM}\times V_{CRM\;}\times \;W_{GM,CRM\;}}{C_{NGM}\times V_{NGM\;}+C_{CRM}\times V_{CRM\;}}$$

*W_RM_*: GM mass fraction of the prepared reference material (in g/kg);

*C_CRM_*: concentration (in copies/µL) of the lec target in the DNA solution extracted from the CRM;

*V_CRM_*: volume (in µL) taken of the DNA solution extracted from the CRM;

*W_GM,CRM_*: certified value for the GM mass fraction of the CRM (in g/kg);

*C_NGM_*: concentration (in copies/µL) of the lec target in the DNA solution extracted from the NGM;

*V_NGM_*: volume (in µL) taken of the DNA solution extracted from the NGM.

Secondly, we estimated the uncertainty associated with the produced reference material considering the individual standard uncertainty contributions for each variable, as described in Trapmann et al. (2013) [32] and in Gatto et al. (2022) [19]:

- Uncertainty of the lec concentration of the DNA solution extracted from the CRM (uc,CRM): estimated as the standard error of the mean of cCRM.
- Uncertainty of the lec concentration of the DNA solution from the non-GM material (uc,NGM): estimated as the standard error of the mean of cNGM. Uncertainty associated with the certified value of the CRM used (uw,CRM): the standard uncertainty of the certified value taken.
- Uncertainty associated with the volume of the taken GM DNA solution (uv,CRM): estimated as the standard error of the pipette volume taken from the technical specification of the pipette.
- Uncertainty associated with the volume of the taken NGM DNA solution (uv,NGM): estimated as the standard error of the pipette volume taken from the technical specification of the pipette.

The combined uncertainty was estimated considering the determination of DNA concentration, the pipetting, the volume mixed and the purity of materials. It was calculated by taking the square root of the sum of squares of these values following the formula [32]

(4) $$\;u=\sqrt{u_{\left(wGM,mGM\right)}2+u_{\left(wGM,pGM\right)}2+u_{\left(wGM,mNGM\right)}2+u_{\left(wGM,pNGM\right)}2\;}$$

- *u*: combined standard uncertainty;
- *u*_(*wGM*,*mGM*)_: standard uncertainty in the function of wGM and the weighed GM material;
- *u*_(*wGM*,*pGM*)_: standard uncertainty in the function of wGM and the purity of GM material;
- *u*_(*wGM*,*mNGM*)_: standard uncertainty in the function of wGM and the weighed non-GM material;
- *u*_(*wGM*,*pNGM*)_: standard uncertainty in the function of wGM and the impurity of non-GM material.

## 3. Results and Discussion

### 3.1. Specificity Evaluation

The formation of unexpected PCR products as dimers or self-dimers among oligonucleotides (primers/probes) cause lower amplification efficiency due to primer depletion and the accumulation of unspecific DNA [7,29,33]. The analysis software used in this work, calculates secondary structures using established methods and reporting changes in Gibbs free energy (ΔG) values. A ΔG value smaller than −9 kcal/mol is an absolute indicator of dimer formation [34]. All of the possible primer/probe combinations were analysed for each duplex, confirming the theoretical specificity of the methods (Table S2A,B). Although a strong ΔG value (–12.8 kcal/mol) for MON87701 oligonucleotides and the lectin forward primer was recorded, no unspecific products were detected experimentally. Indeed, it is crucial to consider the strength of a secondary structure and the position. Rychlik et al. [33] reported how a hairpin loop with negative ΔG at the 3′ end should be avoided since this can cause internal primer extension and a decrease in PCR yield. Hairpins near the 5′ end, however, do not significantly affect the PCR. The specificity was evaluated experimentally too, observing the DNA melt peaks in the post-PCR analysis [31]. No additional peaks were observed, confirming the theoretical specificity of the methods.

### 3.2. Cross-Talk

The experimental design of the cross-talk test was conducted in the absence of each GM event and with 48,270 lectin copies determined for non-modified soybean (AOCS 0906-A) or with 33,696 lectin copies determined for non-modified soybean (AOCS 0911-A) in three replicates per testing condition (Table 1). No cross-talk signal was observed. Gunson et al. [35] reported that in most cases, cross-talk can be eliminated with the appropriate test optimization or PCR platform recalibration. However, this parameter is strictly dependent on the PCR platform and whether the PCR kit uses ROX as a reference dye. In addition, as reported in the “Guidelines for the verification of methods using digital PCR” [36], the usability of fluorophores and quenchers depends on the instrument system and the corresponding instrument settings.

### 3.3. Robustness

Robustness was evaluated at the concentration close to the LOQ, applying some modifications to the original protocol. This parameter was assessed through a multifactorial experiment design [37] using four deliberate modifications: (1) a reduced reaction volume of mastermix (18 µL instead of 20 µL); (2) an increase of 1 °C of the annealing temperature; (3) the use of a slower ramp rate; and (4) decreasing oligonucleotide concentration (see Table 1). An RSDr and bias ≤ 30% are allowed between the copy numbers determined by the original and modified assays for the approach to be considered robust. For all the GM soybean considered, the RSDr and bias were below 30% at the 0.1% GM level (Table 2).

### 3.4. Dynamic Range

The dynamic range was assessed in terms of trueness and precision as reported in the guidelines of ENGL [12,14]. The agreement between the mean of the reference value and the measured one is indicated by trueness, expressed in terms of relative bias, while the precision was evaluated in terms of RSDr obtained under repeatability conditions. The dynamic range for the MON87701, MON87769, MON89788, and CV-127-9 covered the concentrations from 0.1 to 100% and met the acceptance criteria for precision (RSDr ≤ 25%) and trueness (bias ≤ 25%) as showed in Table 3.

### 3.5. Linearity

**Figure 1:**
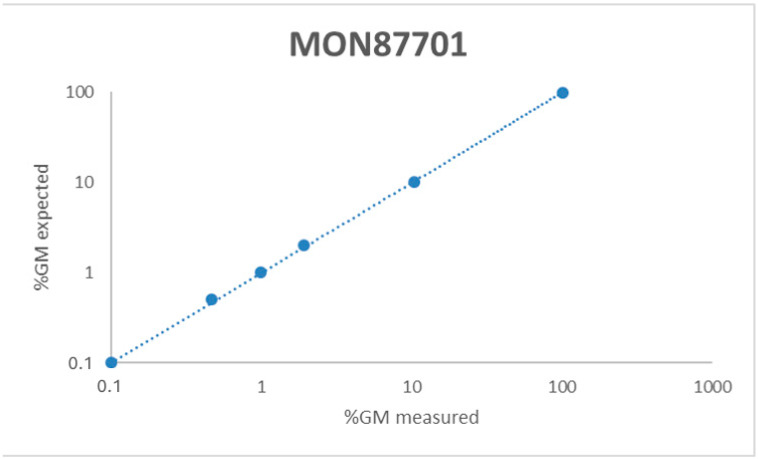

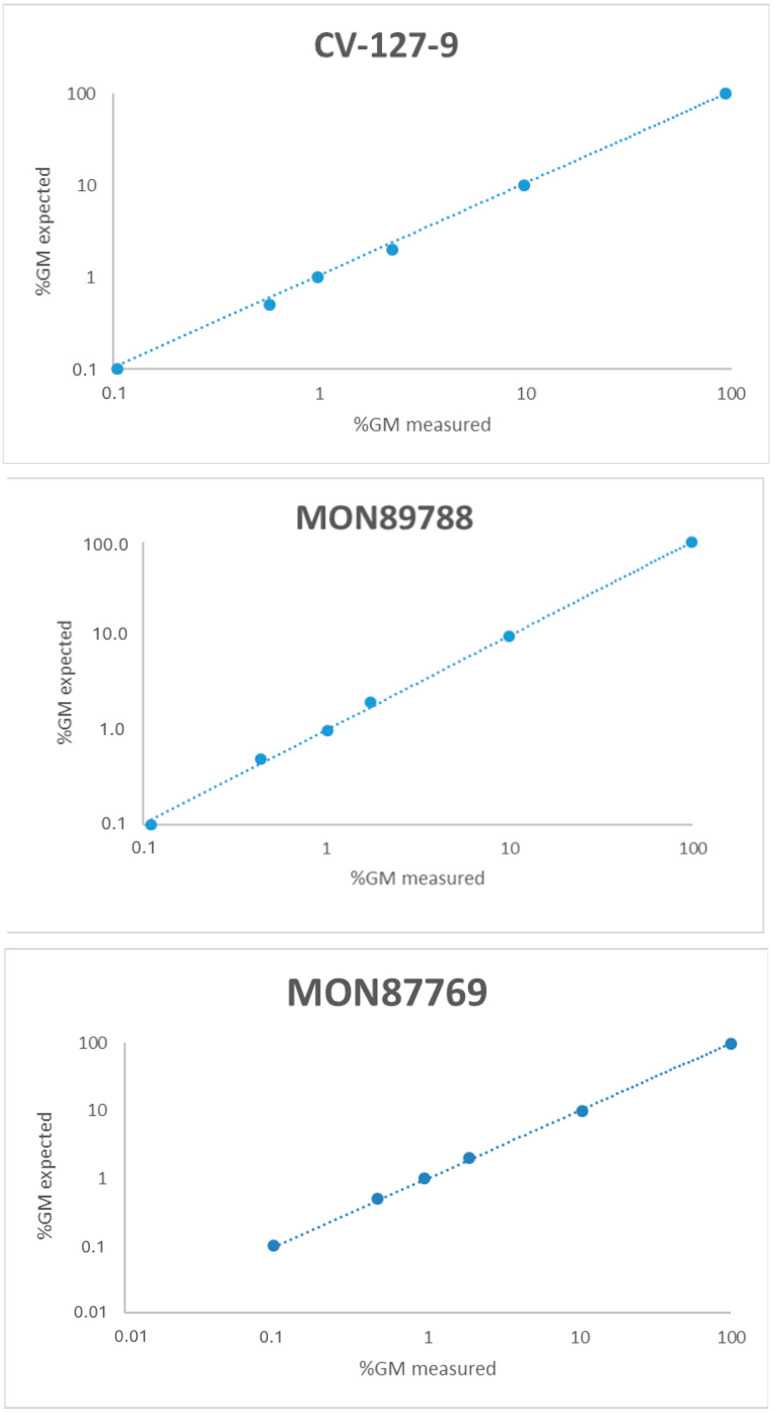
Correlation between measured GM level per reaction of MON87701, MON87769, MON89788, and CV-127-9.

The linearity was evaluated within the dynamic range, observing a directly proportional value to the relative content of the tested GM samples. The degree of linearity within the dynamic range was assessed in terms of the slope and coefficient of determination (R^2^) by a regression analysis of the expected vs. observed GMO content (%) observed for each of the five runs. R^2^ as reported in the ENGL guidelines [12,14] should be greater or equal to 0.98, and the slope should be 1± 0.25. All of the duplex assays fulfil the acceptance criteria, exhibiting an R^2^ of 1 and a slope within the acceptable range (Figure 1 and Table 4).

### 3.6. Trueness

The trueness was evaluated by estimating the relative bias (bias %) for each GM level measured as the relative difference between the average measured sample concentration and the certified sample concentration (in the case of 100% GM) or expected sample concentration (in the case of % GM created in the laboratory). The bias for all the GM events tested was below 25% as established by ENGL’s guidelines [12,14], as reported in Table 5.

In order to investigate if the bias was significant or not, the uncertainty associated with the bias (Ubias) was calculated following the formula reported in the ENGL guidelines [6]. Indeed, as the absolute value of the estimated bias is smaller than Ubias, such a singular bias cannot be considered significant.

### 3.7. Precision

The relative standard deviation of the test results obtained under intermediate repeatability conditions (RSDr) is the parameter used to assess the precision of a quantitative method. A slight decrease in precision is observed for all GM soybeans tested at the 0.1% GM level (Table 5) with an RSDr close to 25%, thus falling within the acceptance criterion. As it can be observed in Table 5, the method satisfies this requirement at all GM levels tested.

### 3.8. Asymmetric Limit of Quantification (LOQ_asym_)

Since each method comprises a duplex GM assay and taxon-specific assay, a low GM content in the context of a high number of taxon-specific targets in asymmetric conditions is represented by the 0.1% GM level. For each GM event, 60 replicates at the 0.1% GM level were tested. The determined LOQ_asym_ was 1.71 copies per reaction for MON87701, 1.23 copies per reaction for MON87769, 1.45 copies per reaction for MON89788, and 1.12 copies per reaction for CV-127-9 (Table S3). These results were compliant with the minimum performance parameters [12,13,14], showing an acceptable level of precision expressed in terms of RSDr (≤25%).

### 3.9. Measurement Uncertainty (MU)

From the same data used for the dynamic range, linearity, trueness, precision (30 data points per each of the six GM levels), MU was estimated. Expanded uncertainty was expressed with a confidence level of 95% (k = 2), as indicated in Table 5.

## 4. Conclusions

In conclusion, in this work, the event-specific methods already validated in a real-time PCR by JRC were transferred (MON87701, MON87769, MON89788, CV-127-9 soybean and lectin reference gene assays) and optimized with the exception of MON87701 in the duplex assay in the ddPCR format. The validation parameter according to the ENGL documents were investigated [12,14]. The obtained results are in compliance with international standards (CCMAS, 2010; ISO, 1994) and in agreement with EU requirements [12,14]. Despite the considerable differences between the *in-house* validation and full validation, the comparison between validation parameters of simplex qPCR GM event-specific methods published by EURL-GMFF [21,22,23,24] and the ddPCR duplex assay values obtained in this study (indicated in Table 6) showed similar results in terms of precision and trueness. Therefore, the methods here presented are suitable for collaborative validation studies and can be a suitable tool in the enforcement of the EU official control legislation, with the duplex ddPCR methods being cost- and time-efficient as long as the use of the conversion factors for the digital PCR analysis of GMO is provided for the transformation of measurement units [10,30,38]. The characteristics of the ddPCR system enable the quantification of GMO content in samples tested in a much lower number of reactions, thus reducing the cost of analysis and decreasing the uncertainty linked to dilution pipetting errors. The development and application of multiplex and screening methods can lower the costs of GMO analysis considerably.

Nonetheless, it was not the purpose of this study to achieve a better performance than that of a qPCR, which is still the gold standard in GMO detection, but rather to show that a ddPCR is comparable in terms of performance parameters. The uncertainty measurement was estimated using CRMs, although it is recommended use real-life samples [25].

## Figures and Tables

**Table 1:** Experimental design for in-house validation of duplex assay ddPCR for lectin/MON87701;lectin/MON87769;Lectin/MON89788 and lectin/CV-127-9.

	EXPERIMENTAL CONDITIONS								DATA	ACCEPTANCE CRITERIA	
INVESTIGATED PARAMETERS	Starting Material	Practical Approach	PCR Assay Design			Copies/Reaction per PCR Mix			Collecting Output and Analysis	Accepted Reference Value	Ref Source
			PCR Replicates	PCR Run	N. Operator						
Specificity (In silico theoretical test)	All oligonucleotides sequences combination for each target	Each duplex system was investigated through alignment with appropriate softwares to assess the generation of no specific PCR products e.g., cross-dimer or self-dimers	N.A.	N.A.	N.A.	N.A.			∆G evaluation and secondary structure generation	Absence of unexpected/unwanted non-target amplification: ∆G ≤9 Kcal/mol	[14]
Experimental specificity (melting curve analysis of the PCR products)	DNA extracted from CRMs: AOCS 0809-A2, AOCS 0809-B2, AOCS 0906-B2, AOCS 0911-C2, AOCS 0906-A, AOCS 0911-A	DNA melting curve analysis using EvaGreen chemistry. qPCR run was performed using pair of oliognucleotides to detect GM event specific and endogeneous gene	2	1	1	N.A.			T melting analysis	No additional peak should be observed	[31]
Cross-talk	DNA extracted from CRMs non-modified soybean: AOCS 0906-A, AOCS 0911-A	Measured the fluorescence signal in presence of an excess of lectin gene and in absence of each GM soybean event	3	1	1	GM gene: 0 copies. Taxon-specific target:—48270 lectin copies determined for AOCS 0906-A.—33696 lectin copies determined for AOCS 0911-A			The fluorescence signals generated during the amplification of different targets evaluation	No cross-talk should be detected, but minimal cross-talk if below the fluorescence threshold is acceptable	[13]
Robustness	DNA extracted from: AOCS 0809-A2, AOCS 0809-B2, AOCS 0906-B2, AOCS 0911-C2	Soybean DNA 0.1% GM (LOQ_asym_ value) prepared considering the practical dilution factor by measuring the content of Lectin reference gene for the GM positive and a GM negative DNA according to the formula described by Hougs et al., 2017, JRC [26]	6 replicates per condition	4 conditions tested, changing of: Temp ramp rate; Annealing temp; Oligont conc; Master mix vol	1	MON87701 0.1% (34.2 cp/rxn), MON87769 0.1% (24.6 cp/rxn), MON89788 0.1% (29 cp/rxn), CV-127-9 0.1% (22.4 cp/rxn)			The copy number for both GM event and lectin gene evaluation	Precision and trueness should not exceed 30% for all combinations	[14]
Dynamic range of the lectin gene	DNA extracted from CRMs non-modified soybean: AOCS 0906-A	A dilution series of conventional soybean at five different lectin copy number (10525, 5262, 2630, 263, 43)	16 replicates per dilution level	5	1	10525 cp/rxn (2100 cp/uL); 5262 cp/rxn (1050 cp/uL); 2630 cp/rxn (526 cp/uL); 263 cp/rxn (53 cp/uL); 43 cp/rxn (9 cp/uL)			R^2^ and slope evaluation between the theoretical and observed values of copy number for lectin gene. Outlier evaluation performed by Grubb’s test	The R^2^ should be ≥0.98% while slope should be 1.00 ± 0.25	[13]
Dynamic range (duplex system for each event)	DNA extracted from: AOCS 0809-A2, AOCS 0809-B2, AOCS 0906-B2, AOCS 0911-C2	GM soybean 0.1%, 0.5%, 1%, 2%, 10% prepared considering the practical dilution factor as described by Hougs et al., 2017, JRC [26]	6 replicates per GM level tested	5 runs 5 days	1	Six content level from 100% to 0.1% GM (0.1%, 0.5%, 1%, 2%, 10%, 100%)			R^2^ and slope evaluation between the theoretical and observed values of GM %. Outlier evaluation performed by Grubb’s test	The R^2^ should be ≥0.98% while slope should be 1.00 ± 0.25	[13]
Linearity						GM target range; 0.1–100% GM			Regression analysis. Outlier evaluation performed by Grubb’s test	The slope of a plot observed vs expected value should be 1.00 ± 0.25 R^2^ ≥ 0.98%	[14]
Trueness (bias)						Six content level from 100% to 0.1% GM (0.1%, 0.5%, 1%, 2%, 10%, 100%) per each level 30 values are considered			Anova one-way and outliers evaluation by Grubb’s test	Bias ≤ 25%	[14]
Precision (RSDr)										RSDr ≤ 25%	[14]
LOQ_asym_		For each event 0.1% GM prepared considering the practical dilution factor as described by Hougs et al., 2017, JRC [26]	≥60 replicates per module	1	1		GM target	Lec	Outlier evaluation performed by Grubb’s test	RSDr ≤ 25% Trueness within a bias of ±25% of the reference value (theorical value of the 0.1% GM prepared in laboratory with 100% GM and non-modified soybean)	[13]
							cp/uL	cp/uL			
						MON87701	1.71	1776			
						MON87769	1.23	1316			
						MON89788	1.45	1411			
						CV-127-9	1.12	1165			

N.A.= not applied.

**Table 2:** Robustness of duplex assay for Lec/GM soybean events; tested 16 conditions at the 0.1% GM level.

Protocol		MON87701			MON87769			MON89788			CV-127-9		
		Mean (%)	RSDr (%)	Bias (%)	Mean (%)	RSDr (%)	Bias (%)	Mean (%)	RSDr (%)	Bias (%)	Mean (%)	RSDr (%)	Bias (%)
Ramp rate change and annealing temp unchanged	Original	0.08	19.1	15.92	0.08	20.15	15.3	0.09	23.4	13.67	0.09	4.7	6.77
	−10% Primer Probe −10% Master Mix	0.09	25.06	11.82	0.09	20.83	7.72	0.08	7.35	23.43	0.1	10.19	2.42
	−10% Primer Probe	0.1	16.92	2.48	0.09	11.15	7.12	0.08	21.06	17.91	0.09	14.02	7.04
	−10% Master Mix	0.1	21.4	0.28	0.1	11.82	0.38	0.07	20.9	25.31	0.09	3.03	6.42
Annealing temp +1 °C	No Change PCR reagents	0.09	13.26	12.62	0.1	26.81	3.49	0.08	7.57	18.27	0.1	22.92	4.56
	−10% Primer Probe −10% Master Mix	0.11	17.7	5.7	0.1	4.38	4.29	0.09	13.99	14.54	0.09	22.08	13.59
	−10% Primer Probe	0.08	10.23	16.3	0.1	24.54	0.28	0.08	7.83	20.29	0.1	26.61	0.24
	−10% Master Mix	0.09	15.88	10.99	0.11	7.37	14.57	0.08	9	21.87	0.11	5.35	11.64
Annealing temp +1 °C and ramp rate + 0.5 °C/s	No Change PCR Reagents	0.08	8.47	15.42	0.09	23.12	12.36	0.08	14.02	22.24	0.11	18.47	14.44
	−10% Primer Probe −10% Master Mix	0.09	2.28	9.97	0.11	5.86	6.52	0.09	21.28	12.33	0.09	15.78	8.63
	−10% Primer Probe	0.08	26.07	16.72	0.08	19.44	16.88	0.08	11.65	23.07	0.10	15.37	0.94
	−10% Master Mix	0.07	16.23	26.55	0.08	12.73	17.12	0.08	16.81	24.81	0.09	15.69	8.86
Ramp rate + 0.5 °C/s	No Change PCR Reagents	0.08	6.66	23.13	0.08	21.42	18.85	0.09	16.92	14.66	0.11	24.38	11.89
	−10% Primer Probe −10% Master Mix	0.08	21.44	15.23	0.08	12.64	15.27	0.08	12.93	21.6	0.08	24.78	16.01
	−10% Primer Probe	0.12	24.48	24.53	0.11	15.35	12.62	0.08	19.05	19.19	0.1	13.76	0.21
	−10% Master Mix	0.1	5.56	2.78	0.08	10.27	19.45	0.08	26.92	23.69	0.12	11.28	20.95

**Table 3:** Dynamic range of GM soybean events expressed in GM-level percentage and relative values of trueness (Bias %) and precision (RSDr).

GM EVENT												
	MON87701			MON87769			MON89788			CV-127-9		
GM Level (%)	Measured Mean (%)	Bias (%)	RSDr (%)	Measured Mean (%)	Bias (%)	RSDr (%)	Measured Mean (%)	Bias (%)	RSDr (%)	Measured Mean (%)	Bias (%)	RSDr (%)
0.1	0.1	0.48	17.82	0.1	3.31	19.1	0.11	11.25	24.27	0.1	4.72	23.39
0.5	0.47	6.44	10.92	0.46	6.5	8.63	0.44	12.19	14.51	0.57	14.53	13.9
1	1	0.41	7.64	0.95	4.67	8.29	1.01	1.3	8.74	0.98	2.01	8.83
2	1.92	4.08	4.91	1.87	6.29	3.67	1.74	13.16	12.72	2.25	12.54	11.49
10	10.38	3.77	3.05	10.49	4.87	3.13	9.96	0.38	3.31	9.9	0.99	1.81
100 ^1^	100.22	1.85	2.04	99.53	0.07	1.66	98.6	1	1.97	93.63	2.77	2.37

^1^ Corresponding to CRM 98.4% MON87701; CRM 99.6% MON87769; CRM 99.6% MON89788; CRM 96.3% CV-127-9.

**Table 4:** Linearity GM-level duplex assay for Lec/GM events (%).

	MON87701			MON89788			MON87769			CV-127-9		
	Slope	Intercept	R^2^	Slope	Intercept	R^2^	Slope	Intercept	R^2^	Slope	Intercept	R^2^
R 1	0.98	−0.01	1.00	1.01	−0.01	1.00	1.02	−0.07	1.00	1.05	−0.16	1.00
R 2	0.98	0.21	1.00	1.01	0.05	1.00	1.00	−0.06	1.00	1.06	−0.24	1.00
R 3	0.99	−0.01	1.00	1.01	0.09	1.00	1.00	−0.07	1.00	1.07	−0.21	1.00
R 4	0.99	−0.03	1.00	1.02	0.03	1.00	1.00	−0.05	1.00	1.06	−0.21	1.00
R 5	0.97	0.05	1.00	1.00	0.05	1.00	0.99	0.004	1.00	1.07	−0.22	1.00
Mean	0.98	0.04	1.00	1.01	0.04	1.00	1.00	−0.05	1.00	1.06	−0.21	1.00

**Table 5:** Performance parameters for the duplex ddPCR assays for each GM event.

	GM Level (%)											
Performance Parameters	MON87701						MON87769					
	0.1	0.5	1	2	10	98.40	0.1	0.5	1	2	10	99.60
N replicates	30	30	30	30	30	30	30	30	30	30	30	30
N outliers	0	0	0	0	0	0	0	0	0	0	0	0
Statistical outlier evaluation	N.A.	N.A.	N.A.	N.A.	N.A.	N.A.	N.A.	N.A.	N.A.	N.A.	N.A.	N.A.
Measured GM content (%)	0.1	0.47	1	1.92	10.38	100.22	0.1	0.46	0.95	1.87	10.49	99.53
Sr	0.018	0.051	0.076	0.094	0.317	2.04	0.018	0.04	0.079	0.12	0.328	1.65
RSDr %	17.82	10.92	7.64	4.91	3.053	2.04	19.1	8.63	8.29	6.34	3.13	1.66
Ubias	0.31	0.48	1.52	3.62	7.52	8.58	0.32	0.38	1.56	5.60	7.35	5.92
Bias	0.0005	0.032	0.004	0.082	0.377	1.82	0.003	0.03	0.05	0.13	0.49	0.07
Bias %	0.48	6.44	0.41	4.08	3.77	1.85	3.31	6.5	4.67	6.29	4.87	0.07
Difference with certified value ^2^	NO	NO	NO	NO	NO	NO	NO	NO	NO	NO	NO	NO
Expanded uncertainty (%, with k = 2)	0.03	0.05	0.15	0.36	0.75	0.91	0.03	0.05	0.16	0.56	0.74	0.81
	**GM level (%)**											
**Performance parameters**	**MON89788**						**CV-127-9**					
	**0.1**	**0.5**	**1**	**2**	**10**	**99.60**	**0.1**	**0.5**	**1**	**2**	**10**	**96.30**
N replicates	30	30	30	30	30	30	30	30	30	30	30	30
N outliers	1	0	0	0	1	1	0	0	0	0	1	0
Statistical outlier evaluation	G ^1^	N.A.	N.A.	N.A.	G ^1^	G ^1^	N.A.	N.A.	N.A.	N.A.	G ^1^	N.A.
Measured GM content (%)	0.11	0.44	1.01	1.74	9.96	98.6	0.1	0.57	0.98	2.25	9.9	93.63
Sr	0.027	0.06	0.089	0.22	0.33	1.94	0.02	0.08	0.09	0.26	0.18	2.22
RSDr %	24.27	14.51	8.74	12.72	3.31	1.97	23.39	13.9	8.83	11.49	1.81	2.37
Ubias	0.38	0.23	1.89	2.66	6.92	3.98	0.6	0.51	3.11	6	16.07	20.15
Bias	0.01	0.06	0.01	0.26	0.04	1	0.004	0.07	0.02	0.25	0.1	2.67
Bias %	11.25	12.19	1.3	13.16	0.38	1	4.72	14.53	2.01	12.54	0.99	2.77
Difference with certified value ^2^	NO	NO	NO	NO	NO	NO	NO	NO	NO	NO	NO	NO
Expanded uncertainty (%, with k = 2)	0.04	0.05	0.19	0.34	0.7	0.53	0.06	0.05	0.31	0.6	1.61	2.22

^1^ G = Grubb’s test. ^2^ The difference in the measurement result and the certified value was considered not significant at the 95% confidence level when Bias ≤ Ubias. N.A.= not applied.

**Table 6:** A comparison between the qPCR and ddPCR results. The qPCR data were obtained from validation reports published by the EURL-GMFF (European Union Reference Laboratory for Genetically Modified Food and Feed). The ddPCR data are presented in this study. The outliers are evaluated through Cochran’s and Grubbs’ tests according to ISO 5725-2 [39].

	MON87701											MON87769										
Performance Parameters	qPCR					ddPCR						qPCR					ddPCR					
	0.085	0.26	0.9	2.7	8.1	0.1	0.5	1.00	2.00	10.00	98.4 ± 0.8	0.1	0.5	0.9	5.0	9.0	0.1	0.5	1.00	2.00	10.00	99.6 ± 0.2
N replicates	48	48	48	48	48	30	30	30	30	30	30	48	48	48	48	48	30	30	30	30	30	30
N outliers	1	1	2	3	2	0	0	0	0	0	0	1	0	2	1	0	0	0	0	0	0	0
Statistical outlier evaluation	1C	1G	1C 1G	1C 2DG	2C	-	-	-	-	-	-	1C	-	2C	1C	-	-	-	-	-	-	-
Measured GM content (%)	0.09	0.28	0.95	2.85	8.11	0.1	0.47	1	1.92	10.38	100	0.09	0.47	0.88	5.2	9.2	0.1	0.46	0.95	1.87	10.49	99.5
Sr	0.02	0.06	0.15	0.4	0.82	0.018	0.05	0.076	0.094	0.317	2.04	0.01	0.07	0.09	0.37	1.25	0.02	0.04	0.08	0.07	0.328	1.65
RSDr %	18	21	15	14	10	17.82	10.9	7.64	4.91	3.053	2.04	13	14	9.7	7	14	19.1	8.63	8.29	3.67	3.13	1.66
Bias	0.007	0.02	0.05	0.15	0.01	0.001	0.03	0.004	0.082	0.377	1.82	−0.01	−0.03	−0.02	0.23	0.16	0	0.003	0.05	0.13	0.49	0.07
Bias %	8.6	6.4	5.2	5.6	0.1	0.48	6.44	0.41	4.08	3.77	1.85	−5.8	−5.2	−2.2	4.6	1.8	3.31	6.5	4.67	6.29	4.87	0.07
	**MON89788**											**CV-127-9**										
**Performance parameters**	**qPCR**					**ddPCR**						**qPCR**					**ddPCR**					
	**0.1**	**0.4**	**0.9**	**4.00**	**8.00**	**0.1**	**0.5**	**1.00**	**2.00**	**10.00**	**99.6 ± 0.2**	**0.09**	**0.3**	**0.9**	**2.5**	**4.5**	**0.1**	**0.5**	**1.00**	**2.00**	**10.00**	**96.3 ± 1.8**
N replicates	48	48	48	48	48	30	30	30	30	30	30	48	48	48	48	48	30	30	30	30	30	30
N outliers	3	1	1	1	1	1	0	0	0	1	1	0	1	0	1	1	0	0	0	0	1	0
Statistical outlier evaluation	2C 1G	1C	1C	1C	1C	1G	-	-	-	1G	1G	-	C	-	C	C	-	-	-	-	1G	-
Measured GM content (%)	0.09	0.38	0.89	4.42	8.22	0.11	0.44	1.01	1.74	9.96	98.6	0.1	0.32	0.92	2.68	4.76	0.1	0.57	0.98	2.25	9.9	93.6
Sr	0.01	0.08	0.13	0.57	0.99	0.027	0.06	0.089	0.22	0.33	1.94	0.01	0.036	0.144	0.19	0.44	0.02	0.08	0.09	0.26	0.18	2.22
RSDr %	16.2	21.7	14.9	12.9	12	24.27	14.5	8.74	12.72	3.31	1.97	11	11	16	7.1	9.2	23.4	13.9	8.83	11.5	1.81	2.37
Bias	−0.01	−0.02	−0.01	0.42	0.22	0.01	0.06	0.01	0.26	0.04	1	0.01	0.02	0.02	0.18	0.26	0	0.07	0.02	0.25	0.1	2.67
Bias %	−14.1	−5	−0.9	10.5	2.8	11.25	12.2	1.3	13.16	0.38	1	8.1	7.8	2.5	7.3	5.9	4.72	14.53	2.01	12.5	0.99	2.77

C = Cochran’ s test, G = Grubb’s test, DG = double Grubb’s test.

## Data Availability

The original contributions presented in the study are included in the article, further inquiries can be directed to the corresponding author.

## Supplementary Materials

The following supporting information can be downloaded at: https://www.mdpi.com/article/10.3390/foods13244011/s1. Table S1: Oligonucleotides’ sequences and concentrations used in this study; Table S2: Specificity in silico evaluation; Table S3: Determination of LOQ_asym_.
