# Supplementary material for: In-House Validation of Four Duplex Droplet Digital PCR Assays to Quantify GM Soybean Events
Source: Foods. 2024 Dec 11;13(24):4011. doi: 10.3390/foods13244011 (PMC11727554; doi:10.3390/foods13244011)
Supplement: Supplementary file 1 [file foods-13-04011-s001.zip › Tab S3.pdf]

Tab S3. Determination of LOQasym of the MON87701, MON87769, MON89788 and CV-127-9 specific ddPCR module express in copies per reaction (cp/μl). Outlier evaluation determined by Grubb's test

Parameters	GM level 0.1 %							
	MON87701/LEC		MON87769/LEC		MON89788/LEC		CV-127-9/LEC	
	GM copy number (cp/μl)	Endogeneous gene copy number (cp/μl)	GM copy number (cp/μl)	Endogeneous gene copy number (cp/μl)	GM copy number (cp/μl)	Endogeneous gene copy number (cp/μl)	GM copy number (cp/μl)	Endogeneous gene copy number (cp/μl)
Copy number	1.71	1776.05	1.23	1316.25	1.45	1411.58	1.12	1165.71
RSDr (%)	19.76		24.39		22.69		23.62	
N.outlier	0		3		0		0	
