# Supplementary material for: In-House Validation of Four Duplex Droplet Digital PCR Assays to Quantify GM Soybean Events
Source: Foods. 2024 Dec 11;13(24):4011. doi: 10.3390/foods13244011 (PMC11727554; doi:10.3390/foods13244011)
Supplement: Supplementary file 1 [file foods-13-04011-s001.zip › Tab S1.pdf]

Tab S1. Items specific for individual methods

Target	Primer and probe	Gene name and Sequence 5'–3'	Final concentration		Reference
			Real-Time PCR	ddPCR	
CV-127-9	SE-127-f4<br>SE-127-r2<br><br>SE-127-p3	5'– AAC AGA AgT TTC CgT TgA gCT TTA AgA C- 3'<br>5' – CAT TCg TAg CTC ggA TCg TgT AC - 3'<br><br>6-FAM - 5' – TTT ggg gAA gCT gTC CCA TgC CC-3' BHQ1	0.4 pmol/µl<br>0.4 pmol/µl<br><br>0.1 pmol/µl	0.4 pmol/µl<br>0.4 pmol/µl<br><br>0.25 pmol/µl	QT-EVE-GM-011 (EURL GMFF) Savini C. et al.,2011;"Event-specific Method for the Quantification of Soybean CV127 Using Real-time PCR - Validation Report and Protocol".
MON89788	MON 89788-F<br>MON 89788-R<br><br>MON 89788-P	5'- TCC CgC TCT AgC gCT TCA AT- 3'<br>5'- TCg AgC Agg ACC TgC AgA A- 3'<br><br>6-FAM 5'-CTg AAg gCg ggA AAC gAC AAT CTg- 3' BHQ1	0.15 pmol/µl<br>0.15 pmol/µl<br><br>0.05 pmol/µl	0.6 pmol/µl<br>0.6 pmol/µl<br><br>0.25 pmol/µl	QT-EVE-GM-006 (EURL GMFF) Charles Delobel C., Bogni A., Pinski G., Mazzara M., Van Den Eede G. 2008 "Event-specific Method for the Quantification of Soybean Line MON 89788 - Validation Report and Protocol".
MON87701	MON 87701 2<br>MON 87701 1<br><br>MON 87701 P	5' – Tgg TgA TAT gAA gAT ACA TgC TTA gCA T – 3'<br>5' – CgT TTC CCg CCT TCA gTT TAA A – 3'<br><br>6- FAM 5'-TCA gTg TTT gAC ACA CAC ACT AAg CgT gCC-3' BHQ1	0.6 pmol/µl<br>0.6 pmol/µl<br><br>0.25 pmol/µl	0.6 pmol/µl<br>0.6 pmol/µl<br><br>0.25 pmol/µl	QT-EVE-GM-010 (EURL GMFF) Charels D. et al.,2011 "Event-specific Method for the Quantification of Soybean MON87701 Using Real-time PCR - Validation Report and Protocol".
MON87769	MON 87769 F<br>MON 87769 R<br><br>MON 87769 P	5' – CAT ACT CAT TgC TgA TCC ATg TAg ATT - 3'<br>5' – gCA AgT TgC TCg TgA AgT TTT g - 3'<br><br>6-FAM 5' – CCC ggA CAT gAA gCC ATT TAC AAT TgA C – 3' BHQ1	0.6 pmol/µl<br>0.6 pmol/µl<br><br>0.2 pmol/µl	0.6 pmol/µl<br>0.6 pmol/µl<br><br>0.25 pmol/µl	QT-EVE-GM-002 (EURL GMFF) Savini C.,2012 "Event-specific Method for the Quantification of Soybean MON87769 Using Real-time PCR - Validation Report and Protocol".
LECTIN	Lec F<br>Lec R	5'- CCA gCT TCg CCg CTT CCT TC –3'<br>5'- gAA ggC AAg CCC ATC TgC AAg CC –3'	0.3 pmol/µl<br>0.3 pmol/µl	0.6 pmol/µl<br>0.6 pmol/µl	QT-TAX-GM-002 (EURL GMFF)

	Lec P	HEX 5’- CTT CAC CTT CTA TgC CCC TgA CAC 3’BHQ1	0.1 pmol/µl	0.25 pmol/µl	Mazzara M, Charles Delobel C, Pinski G, Savini C, Van den Eede G. 2012. "Event-specific Method for the Quantification of Soybean MON87769 Using Real-time PCR. Validation Report and Validated Method.
