# Supplementary material for: In-House Validation of Four Duplex Droplet Digital PCR Assays to Quantify GM Soybean Events
Source: Foods. 2024 Dec 11;13(24):4011. doi: 10.3390/foods13244011 (PMC11727554; doi:10.3390/foods13244011)
Supplement: Supplementary file 1 [file foods-13-04011-s001.zip › Tab S2.pdf]

Tab S2 A) Outcome of self-dimer evaluation by Primer-dimer software B) Outcome of cross-dimer evaluation by Oligo-evaluator software.

A

ddPCR duplex assay	Forward Primer Name	Forward Primer Seq	Reverse Primer Name	Reverse Primer Seq	Structure	ΔG
MON87701 /Lec	MON 87701 1	CGTTTCCCGCCTTCAGTTTAAA	MON 87701 2	TGGTGATATGAAGATACATGCTTAGCAT	hetero dimer	**-12.88**
	MON 87701 1	CGTTTCCCGCCTTCAGTTTAAA	MON 87701 P	TCAGTGTTTGACACACACACTAAGCGTGCC	hetero dimer	-2.25
	MON 87701 2	TGGTGATATGAAGATACATGCTTAGCAT	MON 87701 P	TCAGTGTTTGACACACACACTAAGCGTGCC	hetero dimer	**-12.88**
	Lec F	CCAGCTTCGCCGCTTCCTTC	Lec P	CTTCACCTTCTATGCCCCTGACAC	hetero dimer	0
	Lec R	GAAGGCAAGCCCATCTGCAAGCC	Lec P	CTTCACCTTCTATGCCCCTGACAC	hetero dimer	-5.28
	Lec F	CCAGCTTCGCCGCTTCCTTC	Lec R	GAAGGCAAGCCCATCTGCAAGCC	hetero dimer	-5.28
	Lec F	CCAGCTTCGCCGCTTCCTTC	MON 87701 2	TGGTGATATGAAGATACATGCTTAGCAT	hetero dimer	**-12.88**
	MON 87701 1	CGTTTCCCGCCTTCAGTTTAAA	Lec R	GAAGGCAAGCCCATCTGCAAGCC	hetero dimer	-5.28
	MON 87701 1	CGTTTCCCGCCTTCAGTTTAAA	Lec P	CTTCACCTTCTATGCCCCTGACAC	hetero dimer	-2.25
MON87769 /Lec	MON 87769 F	CATACTCATTGCTGATCCATGTAGATT	MON 87769 R	GCAAGTTGCTCGTGAAGTTTTG	hetero dimer	0
	MON 87769 F	CATACTCATTGCTGATCCATGTAGATT	MON 87769 P	CCCGGACATGAAGCCATTTACAATTGAC	hetero dimer	-3.01
	MON 87769 F	CATACTCATTGCTGATCCATGTAGATT	Lec F	CCAGCTTCGCCGCTTCCTTC	hetero dimer	0
	MON 87769 F	CATACTCATTGCTGATCCATGTAGATT	Lec R	GAAGGCAAGCCCATCTGCAAGCC	hetero dimer	-5.28
	MON 87769 F	CATACTCATTGCTGATCCATGTAGATT	Lec P	CTTCACCTTCTATGCCCCTGACAC	hetero dimer	0

	MON 87769 F	GCAAGTTGCTCGTGAAGTTTTG	MON 87769 P	CCCGGACATGAAGCCATTTACAATTGAC	hetero dimer	-0.09
	Lec F	CCAGCTTCGCCGCTTCCTTC	Lec R	GAAGGCAAGCCCATCTGCAAGCC	hetero dimer	-5.28
	Lec F	CCAGCTTCGCCGCTTCCTTC	Lec P	CTTCACCTTCTATGCCCCTGACAC	hetero dimer	0
	Lec R	GAAGGCAAGCCCATCTGCAAGCC	Lec P	CTTCACCTTCTATGCCCCTGACAC	hetero dimer	-5.28
MON89788 /Lec	MON 89788-F	TCCCGCTCTAGCGCTTCAAT	MON 89788-R	TCGAGCAGGACCTGCAGAA	hetero dimer	-1.32
	MON 89788-F	TCCCGCTCTAGCGCTTCAAT	MON 89788-P	CTGAAGGCGGGAAACGACAATCTG	hetero dimer	0
	MON 89788-R	TCGAGCAGGACCTGCAGAA	MON 89788-P	CTGAAGGCGGGAAACGACAATCTG	hetero dimer	-4.62
	Lec F	CCAGCTTCGCCGCTTCCTTC	Lec P	CTTCACCTTCTATGCCCCTGACAC	hetero dimer	0
	Lec R	GAAGGCAAGCCCATCTGCAAGCC	Lec P	CTTCACCTTCTATGCCCCTGACAC	hetero dimer	-5.28
	Lec F	CCAGCTTCGCCGCTTCCTTC	Lec R	GAAGGCAAGCCCATCTGCAAGCC	hetero dimer	-5.28
	Lec F	CCAGCTTCGCCGCTTCCTTC	MON 89788-R	TCGAGCAGGACCTGCAGAA	hetero dimer	-5.37
	MON 89788-F	TCCCGCTCTAGCGCTTCAAT	Lec R	GAAGGCAAGCCCATCTGCAAGCC	hetero dimer	-5.28
	MON 89788-F	TCCCGCTCTAGCGCTTCAAT	Lec P	CTTCACCTTCTATGCCCCTGACAC	hetero dimer	0
CV-127-9/Lec	SE-127-f4	AACAGAAGTTTCCGTTGAGCTTTAAGAC	SE-127-r2	CATTCGTAGCTCGGATCGTGTAC	hetero dimer	-7.89
	SE-127-f4	AACAGAAGTTTCCGTTGAGCTTTAAGAC	SE-127-p3	TTTGGGGAAGCTGTCCCATGCCC	hetero dimer	-3.79
	SE-127-r2	CATTCGTAGCTCGGATCGTGTAC	SE-127-p3	TTTGGGGAAGCTGTCCCATGCCC	hetero dimer	-7.89
	Lec F	CCAGCTTCGCCGCTTCCTTC	Lec R	GAAGGCAAGCCCATCTGCAAGCC	hetero dimer	-5.28

	Lec F	CCAGCTTCGCCGCTTCCTTC	Lec P	CTTCACCTTCTATGCCCCTGACAC	hetero dimer	0
	Lec F	CCAGCTTCGCCGCTTCCTTC	SE-127-f4	AACAGAAGTTTCCGTTGAGCTTTAAGAC	hetero dimer	0
	Lec F	CCAGCTTCGCCGCTTCCTTC	SE-127-r2	CATTCGTAGCTCGGATCGTGTAC	hetero dimer	-7.89
	Lec R	GAAGGCAAGCCCATCTGCAAGCC	SE-127-f4	AACAGAAGTTTCCGTTGAGCTTTAAGAC	hetero dimer	-5.28
	Lec P	CTTCACCTTCTATGCCCCTGACAC	SE-127-f4	AACAGAAGTTTCCGTTGAGCTTTAAGAC	hetero dimer	0

B

Name	Sequence	Base Count	Length (bp)	Molecular Weight	Tm (°C)	Extinction Coefficient	µg/OD at 260 nm	GC %	GC Clamp	Run Length (bp)	Primer Dimer	Secondary Structure
MON 87701 1	CGTTTCCCGCCTTCAGTTTAAA	A = 4, U = 0, G = 3, C = 7, T = 8, I = 0, Total = 22	22	6636.4	67.7	198.7	33.4	45.5	0	3	No	None
MON 87701 2	TGGTGATATGAAGATACATGCTTAGCAT	A = 9, U = 0, G = 7, C = 3, T = 9, I = 0, Total = 28	28	8666.8	66.1	283.4	30.6	35.7	2	2	No	**Moderate**
MON 87701 P	TCAGTGTTTGACACACACACTAAGCGTGCC	A = 8, U = 0, G = 6, C = 9, T = 7, I = 0, Total = 30	30	9151.1	76.1	284.2	32.2	50.0	3	3	No	**Moderate**
Lec F	CCAGCTTCGCCGCTTCCTTC	A = 1, U = 0, G = 3, C = 10, T = 6, I = 0, Total = 20	20	5956.0	72.3	162.1	36.7	65.0	2	2	No	Weak
Lec R	GAAGGCAAGCCCATCTGCAAGCC	A = 7, U = 0, G = 6, C = 8, T = 2, I = 0, Total = 23	23	7027.7	75.3	220.5	31.9	60.9	3	3	No	**Moderate**
Lec P	CTTCACCTTCTATGCCCCTGACAC	A = 4, U = 0, G = 2, C = 11, T = 7, I = 0, Total = 24	24	7159.8	68.7	206.1	34.7	54.2	1	4	No	Very Weak
MON 87769 F	CATACTCATTGCTGATCCATGTAGATT	A = 7, U = 0, G = 4, C = 6, T = 10, I = 0, Total = 27	27	8224.5	64.8	257.5	31.9	37.0	1	2	No	Very Weak
MON 87769 R	GCAAGTTGCTCGTGAAGTTTTG	A = 4, U = 0, G = 7, C = 3, T = 8, I = 0, Total = 22	22	6796.5	65.8	209.1	32.5	45.5	1	4	No	Weak

MON 87769 P	CCCGGACATGAAGCCATTTACAATTGAC	A = 9, U = 0, G = 5, C = 8, T = 6, I = 0, Total = 28	28	8541.7	74.0	271.1	31.5	46.4	1	3	No	Very Weak
Lec F	CCAGCTTCGCCGCTTCCTTC	A = 1, U = 0, G = 3, C = 10, T = 6, I = 0, Total = 20	20	5956.0	72.3	162.1	36.7	65.0	2	2	No	Weak
Lec R	GAAGGCAAGCCCATCTGCAAGCC	A = 7, U = 0, G = 6, C = 8, T = 2, I = 0, Total = 23	23	7027.7	75.3	220.5	31.9	60.9	3	3	No	**Moderate**
Lec P	CTTCACCTTCTATGCCCCTGACAC	A = 4, U = 0, G = 2, C = 11, T = 7, I = 0, Total = 24	24	7159.8	68.7	206.1	34.7	54.2	1	4	No	Very Weak
MON 89788-F	TCCCGCTCTAGCGCTTCAAT	A = 3, U = 0, G = 3, C = 8, T = 6, I = 0, Total = 20	20	6004.0	68.1	174.1	34.5	55.0	1	3	No	Very Weak
MON 89788-R	TCGAGCAGGACCTGCAGAA	A = 6, U = 0, G = 6, C = 5, T = 2, I = 0, Total = 19	19	5846.9	67.5	189.7	30.8	57.9	1	2	No	Very Weak
MON 89788-P	CTGAAGGCGGGAAACGACAATCTG	A = 8, U = 0, G = 8, C = 5, T = 3, I = 0, Total = 24	24	7435.9	73.1	240.2	31.0	54.2	1	3	No	Very Weak
Lec F	CCAGCTTCGCCGCTTCCTTC	A = 1, U = 0, G = 3, C = 10, T = 6, I = 0, Total = 20	20	5956.0	72.3	162.1	36.7	65.0	2	2	No	Very Weak
Lec R	GAAGGCAAGCCCATCTGCAAGCC	A = 7, U = 0, G = 6, C = 8, T = 2, I = 0, Total = 23	23	7027.7	75.3	220.5	31.9	60.9	3	3	No	Very Weak
Lec P	CTTCACCTTCTATGCCCCTGACAC	A = 4, U = 0, G = 2, C = 11, T = 7, I = 0, Total = 24	24	7159.8	68.7	206.1	34.7	54.2	1	4	No	Very Weak
SE-127-f4	AACAGAAGTTTCCGTTGAGCTTTAAGAC	A = 9, U = 0, G = 6, C = 5, T = 8, I = 0, Total = 28	28	8611.7	67.1	278.0	31.0	39.3	1	3	No	Very Weak
SE-127-r2	CATTCGTAGCTCGGATCGTGTAC	A = 4, U = 0, G = 6, C = 6, T = 7, I = 0, Total = 23	23	7030.7	66.6	216.7	32.4	52.2	1	2	Yes	Very Weak
SE-127-p3	TTTGGGGAAGCTGTCCCATGCCC	A = 3, U = 0, G = 7, C = 7, T = 6, I = 0, Total = 23	23	7031.6	77.1	207.4	33.9	60.9	4	4	No	**Strong**
Lec F	CCAGCTTCGCCGCTTCCTTC	A = 1, U = 0, G = 3, C = 10, T = 6, I = 0, Total = 20	20	5956.0	72.3	162.1	36.7	65.0	2	2	No	Weak
Lec R	GAAGGCAAGCCCATCTGCAAGCC	A = 7, U = 0, G = 6, C = 8, T = 2, I = 0, Total = 23	23	7027.7	75.3	220.5	31.9	60.9	3	3	No	**Moderate**
Lec P	CTTCACCTTCTATGCCCCTGACAC	A = 4, U = 0, G = 2, C = 11, T = 7, I = 0, Total = 24	24	7159.8	68.7	206.1	34.7	54.2	1	4	No	Very Weak
